# Supplementary figures and images for: Multiplex shRNA Screening of Germ Cell Development by in Vivo Transfection of Mouse Testis
Source: G3 (Bethesda). 2016 Nov 15;7(1):247–55. doi: 10.1534/g3.116.036087 (PMC5217113; doi:10.1534/g3.116.036087)

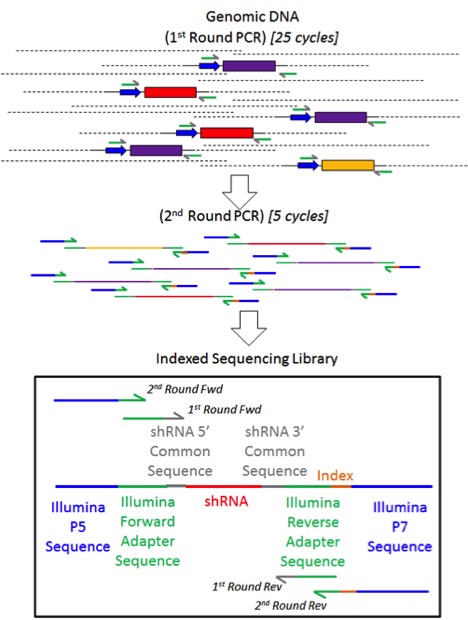

Supplement: Supplementary file 1 [file 247FigureS1.jpg]

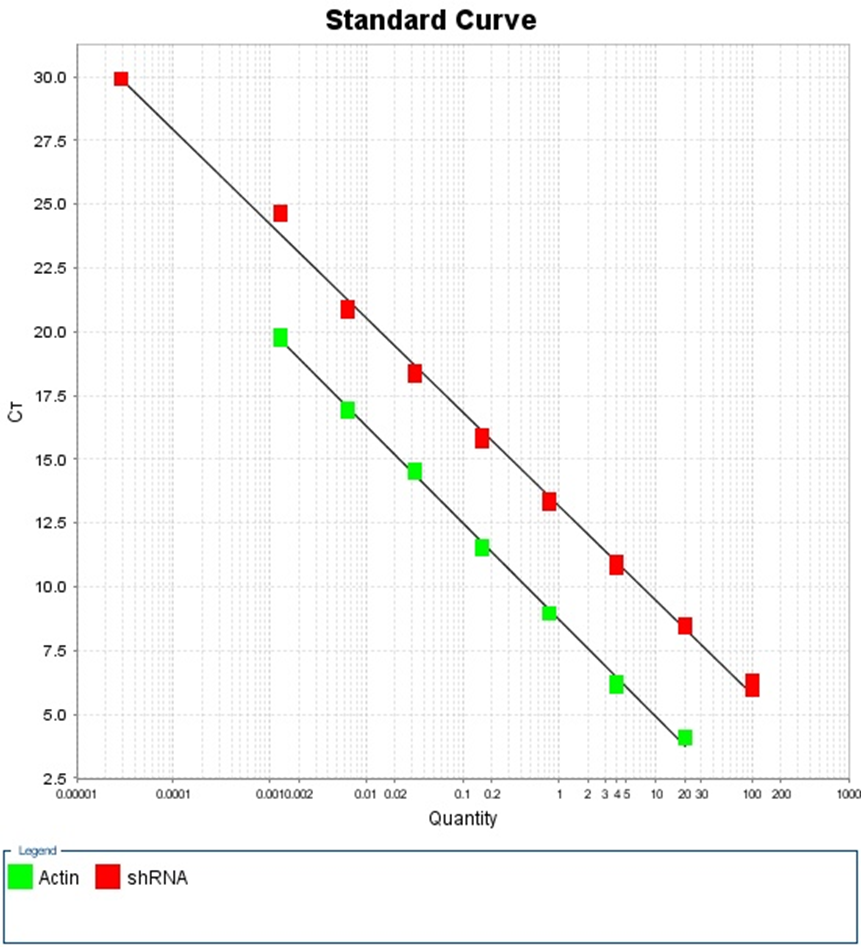

Supplement: Supplementary file 2 [file 247FigureS2.png]

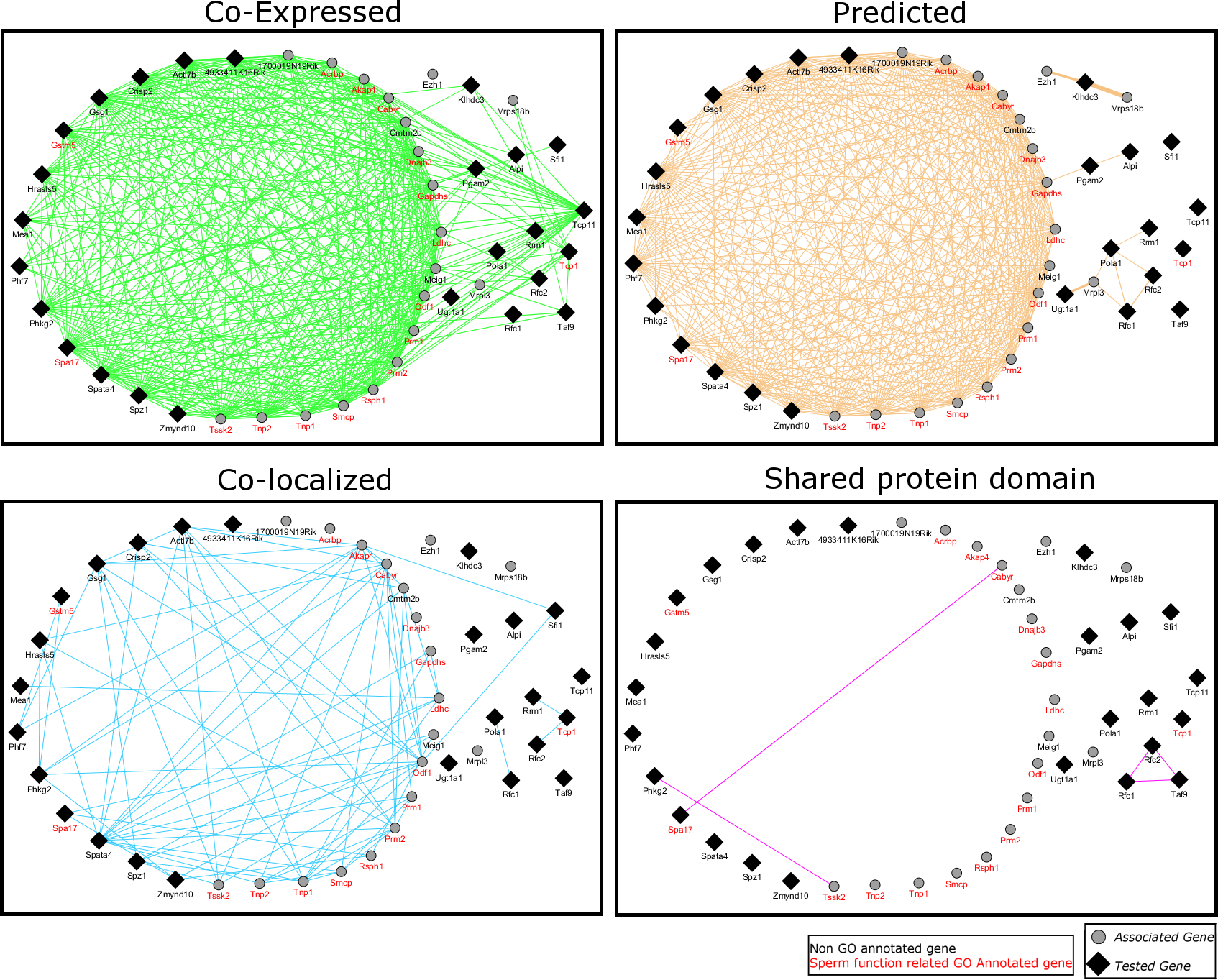

Supplement: Supplementary file 3 [file 247FigureS3.png]
