# Supplementary material for: Multiplex shRNA Screening of Germ Cell Development by in Vivo Transfection of Mouse Testis
Source: G3 (Bethesda). 2016 Nov 15;7(1):247–55. doi: 10.1534/g3.116.036087 (PMC5217113; doi:10.1534/g3.116.036087)
Supplement: Supplementary file 4 [file 247FileS1.docx]

**Mouse Testis DNA injection**

Materials Needed:

- 1M Tris-Hcl pH7.0
- Pure, DNase and RNase free water
- DNA to be injected
- Anesthesia*
- 29 gauge Insulin Needle (Terumo: SS10M2913)
- 701N Syringe, Cemented Needle, 26s Gauge (Hamilton: 80300)
- 70% Ethanol (denatured is fine)
- 100% pure Ethanol
- Kimwipes

**Step 1: Prepare the DNA mixture**

*If using linear DNA*

Dilute the DNA in Tris-Hcl and water to get 15µg of DNA in 20µl of **150mM** Tris-HCL pH 7.0

*If using circular DNA*

Dilute the DNA in Tris-Hcl and water to get 15µg of DNA in 20µl of **125mM** Tris-HCL pH 7.0

**Step 2: Anesthetize the mouse**

We used a mixture of *(final concentrations)* Ketamine (10mg/ml), Xylazine (1mg/ml), and Glycopyrrolate (2µg/ml) diluted in sterile PBS. This was injected intraperitoneally using a 29 gauge needle, with 10µl of anesthesia used per 1g of mouse body weight.

*Alternative general anesthesia methods such as isoflurane can also be used*

**Step 3: Injection of material**

First, wipe down the inferior torso (where the testis are) with 70% ethanol and a kimwipe. (This flattens the fur and prevents it from interfering with the injection.

Nest, feel for one of the mouse testes and get a good grip on it between your fingers.

Using the 701N syringe, pipette 10µl of the DNA mixture form step 1 and inject it slowly through the skin into the anterior end of the testis. (This should take between 30s to 60s to finish injecting. We find that slower rates of injection lead to better transfection rates) Repeat this with the other 10µl using the same syringe into the posterior end of the testis.

Repeat the same procedure for the other testis.

Following Injection, tap the testes gently about ten times with your finger to ensure that the DNA mixture is spread throughout.

At the end, clean the syringe by pulling up 100% ethanol through it three times and wiping down the needle using a kimwipe and 100% ethanol.

*To prevent contamination, we like to use different 701N syringes for different DNA mixtures. However, the same syringe can be used on multiple different testes if you are using an identical DNA mixture for all of them.*

*We space our injections 3-4 days apart (twice a week) to allow the testis to heal from the prior injection.*

*
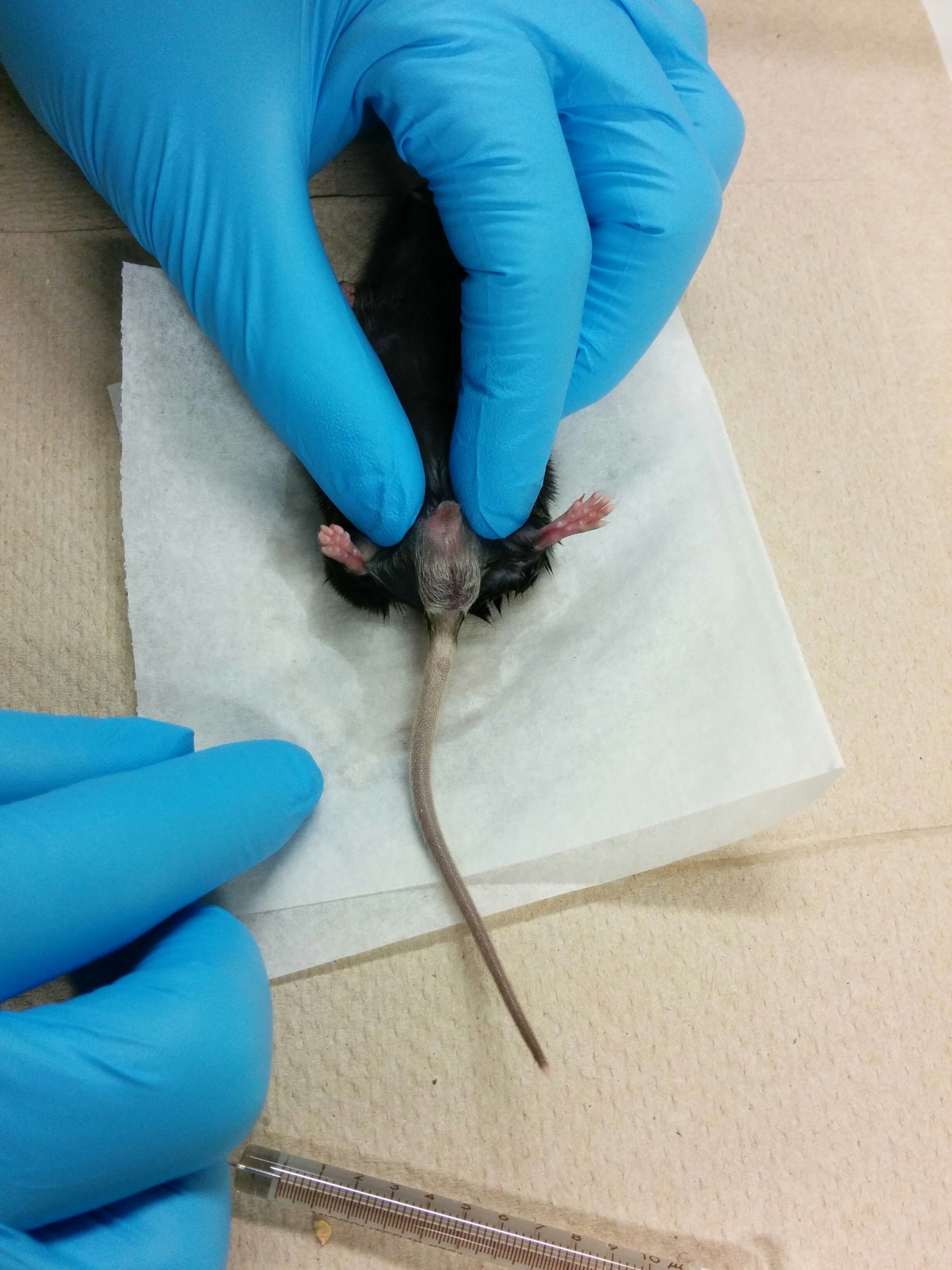
*

**Presenting the testes *(yellow arrows)* for injection**

*
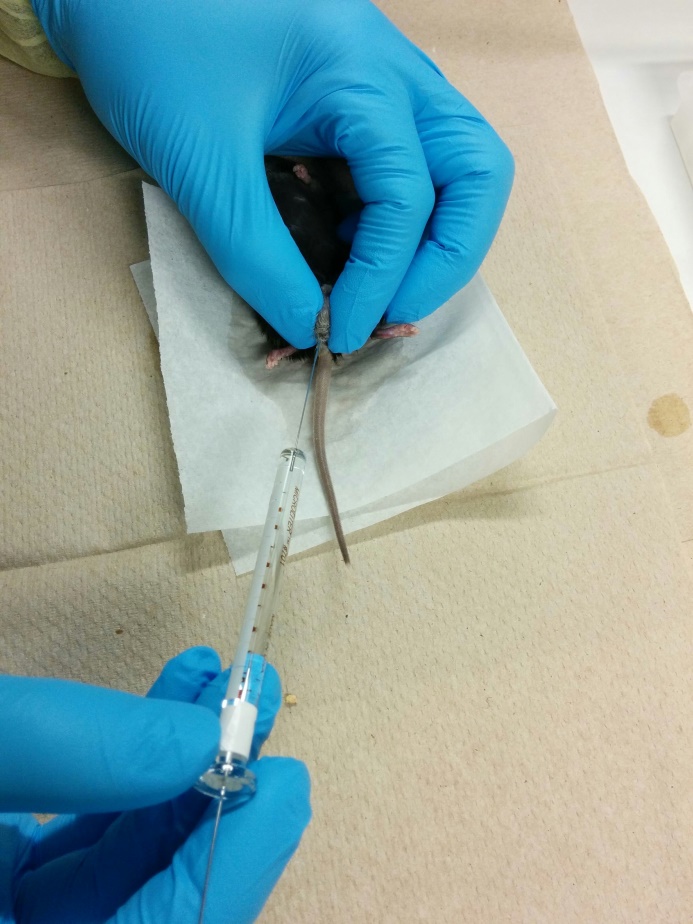

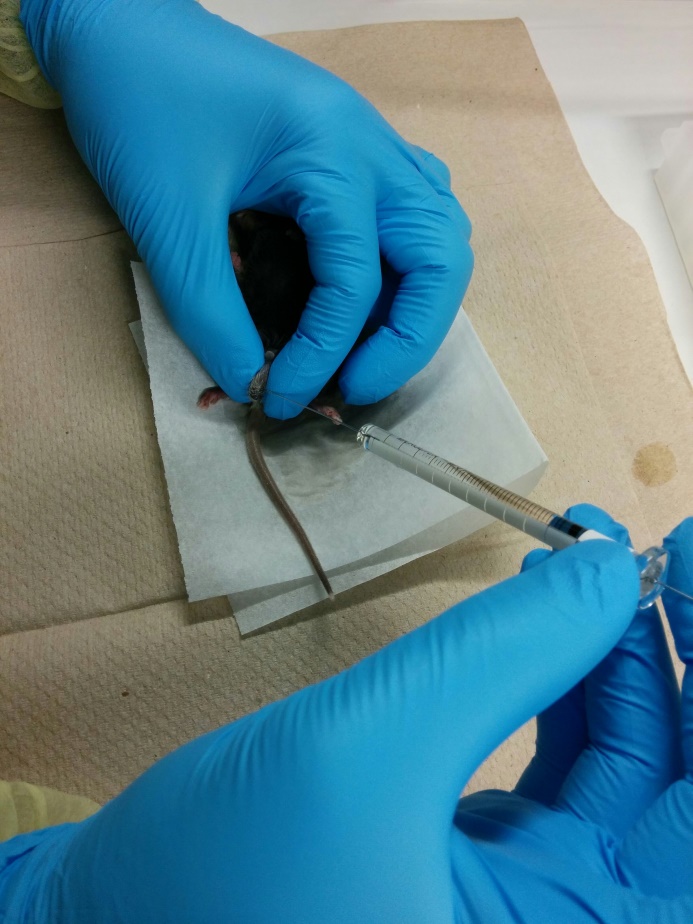
*

**Anterior Injection Posterior Injection**
