# Supplementary material for: Multiplex shRNA Screening of Germ Cell Development by in Vivo Transfection of Mouse Testis
Source: G3 (Bethesda). 2016 Nov 15;7(1):247–55. doi: 10.1534/g3.116.036087 (PMC5217113; doi:10.1534/g3.116.036087)
Supplement: Supplementary file 5 [file 247FileS2.docx]

**shRNA pool DNA Preparation (For Injection)**

Materials Needed:

- Q5 Hot Start DNA polymerase* [NEB: M0493L]
- 5M Betaine [Sigma-Aldrich: B0300-1VL/ B0300-5VL]
- 10mM dNTPs [Promega: U1511/U1515]
- shRNA pool [Sigma-Aldrich: Mission® shRNA Library]
- PCR Primers: *(5’ to 3’)*

| pLKO U6 shRNA F | GAGGGCCTATTTCCCATGATTCC |
| --- | --- |
| pLKO U6 shRNA R | GTGGATGAATACTGCCATTTGTCTC |

- Ampure XP beads [Beckman Coulter: A63880/A63881/A63882]
- 70% Ethanol
- Magnetic separation rack for 1.5ml microcentrifuge tubes

**(We have found Q5 to be more sensitive and specific than Taq or Phusion, and this improvement is necessary for amplifying from low amounts of sample.)*

**Step 1: Mix shRNA pool**

We ordered shRNAs in plasmid form in the 96-well plate format. Taking 5µl of the plasmid each shRNA we wanted in the pool we made a pool of over a hundred shRNAs at a final concentration of around 20ng/µl. This was mixed via a 10 second vortex and spun down briefly to collect all the liquid at the bottom of the tube.

**Step 2: PCR Amplification of shRNA pool**

*Each PCR reaction produces ~ 3-4µg of DNA, meaning around 20 reactions are needed for enough material for 1 testis (15µg/injection X 5 injections = 75µg).*

Per reaction, mix:

| 10μM F and R Primers | 0.4μM | 2μl |
| --- | --- | --- |
| 5X Q5 Buffer | 1X | 10μl |
| 10mM dNTPs | 0.2mM | 1µl |
| Q5 Hotstart Polymerase | 4 Units | 2µl |
| 5M Betaine | 1.5M | 15µl |
| DNA sample | ~20ng | 1µl |
| Distilled H_2_O |  | 19μl |
|  |  | **50µl** |

PCR Conditions

98°C for 3 minutes

98°C for 30 seconds

60°C for 30 seconds 35 Cycles

72°C for 30 seconds

72°C for 10 minutes

4°C hold

*Expected product size is 343bp*

**Step 3: Clean-up of PCR products**

*(Adapted from AMpure XP manufacturer’s protocol)*

1. Pool up to 10 reactions in a 1.5ml microcentrifuge tube.
2. Add 1.8X volume of AMpureXP beads to the mixture (i.e. 900µl beads for a 500µl reaction mix) and incubate for 5 minutes at room temperature
3. Place tubes in the magnetic separation rack for at least 1 minute.
4. Without disturbing the beads on the side of the tube, pipette out the liquid leaving up to 20µl behind.
5. While still on the rack, add 1ml of 70% ethanol to each tube to wash the beads.
6. Incubate for 1 minute at room temperature.
7. Pipette out all the liquid from each tube.
8. Repeat Steps 5 to 7.
9. Air dry the beads for 2 minutes at room temperature
10. Remove tubes from rack and add 200µl of water/elution buffer to the beads, pipetting up and down to ensure all the beads are suspended. Sequentially take the same 200µl bead/water mixture and pipette into the other tubes, until there are 6 tubes worth of beads suspended in the water.
11. Incubate at room temperature for a minimum of 5 minutes.
12. Place tubes in magnetic separation rack and let it sit for 5 minutes.
13. Without disturbing the beads on the side of the tube, pipette out the 200µl of liquid and put into a new tube.
14. Quantify the concentration of DNA using a spectrophotometer.

**shRNA pool Sequencing Library Preparation**

Materials Needed:

- Q5 Hot Start DNA polymerase* [NEB: M0493L]
- 5M Betaine [Sigma-Aldrich: B0300-1VL/ B0300-5VL]
- 10mM dNTPs [Promega: U1511/U1515]
- DNA *(input/genomic)*
- PCR Primers: *(5’ to 3’)*

*(Step 1 PCR primers)*

| pLKO F | CTCTTTCCCTACACGACGCTCTTCCGATCT NNNN CTTTATATATCTTGTGGAAAGGACGA |
| --- | --- |
| pLKO R | CTGGAGTTCAGACGTGTGCTCTTCCGATCT NNNNNN TGGATGAATACTGCCATTTGTCTC |

*Note: NNNN stands for four and NNNNNN stands for six random nucleotides. These are a mixture of 25% of each base and are required to avoid QC errors for Illumina sequencing since the library has a low complexity at the non-shRNA regions.*

*(Step 2 PCR primers)*

| PE PCR F | AATGATACGGCGACCACCGAGATCTAC ACTCTTTCCCTACACGACGCTCTTCCGATCT |
| --- | --- |
| PE PCR R | CAAGCAGAAGACGGCATACGAGAT XXXXXXXX GTGACTGGAGTTCAGACGTGTGCTCTTCCG |

*Note: XXXXXXXX stands for the reverse complement of the index sequence used for multiplexing. This can be between 6-8 base pairs in length. Different R primers can be ordered and used with the F primer. If double indexing, add the reverse complement of the second index to the space in the F primer.*

- (Optional) Qiagen Minelute Kit [Qiagen: 28004]
- Ampure XP beads [Beckman Coulter: A63880/A63881/A63882]
- 70% Ethanol
- 96-well magnetic separation plate

**(We have found Q5 to be more sensitive and specific than Taq or Phusion, and this improvement is necessary for amplifying from low amounts of sample.)*

**Step 1: PCR amplification of shRNA sequences from sample**

Per reaction, mix:

| 10μM pLKO F and R primers | 0.4μM | 1μl |
| --- | --- | --- |
| 5X Q5 Buffer | 1X | 5μl |
| 10mM dNTPs | 0.2mM | 0.5µl |
| Q5 Hotstart Polymerase | 2 Units | 1µl |
| 5M Betaine | 1.5M | 7.5µl |
| Genomic DNA sample | ~2µg | X µl |
| Distilled H_2_O |  | 10-X µl |
|  |  | **25µl** |

PCR Conditions

98°C for 3 minutes

98°C for 30 seconds

60°C for 30 seconds 25 Cycles

72°C for 30 seconds

72°C for 10 minutes

4°C hold

**Note: If amplifying from input DNA pool, use ~100ng of sample.*

*Expected product size is 196bp*

**(Optional) Step 2: Purification of DNA sample**

*This step removes excess primers which can inhibit the PCR reaction in step 3*

Use the Qiagen Minelute kit with the manufacturer’s protocol to purify, eluting the sample in 10µl of EB.

Alternative: Use the AMpure XP beads to purify the sample similar to step 4, scaling the volume of beads added to the 25µl PCR reaction volume (45µl of beads) and eluting in 20µl of water/elution buffer.

**Step 3: Illumina Library Preparation using PCR**

Per reaction, mix:

| 10μM PE PCR F and R primers | 0.4μM | 2μl |
| --- | --- | --- |
| 5X Q5 Buffer | 1X | 10μl |
| 10mM dNTPs | 0.2mM | 1µl |
| Q5 Hotstart Polymerase | 4 Units | 2µl |
| 5M Betaine | 1.5M | 15µl |
| Step 1 DNA sample | 200-500ng | X µl |
| Distilled H_2_O |  | 20-X µl |
|  |  | **50µl** |

PCR Conditions

98°C for 3 minutes

98°C for 30 seconds

60°C for 30 seconds 6 Cycles

72°C for 30 seconds

72°C for 10 minutes

4°C hold

**Note: If planning to run different samples in the same sequencing lane, ensure that the F and R primer combination of indices are different for each sample so that you can demultiplex during analysis.*

*If skipping Step 2, X should be half of the volume of the step 1 reaction (12.5µl)*

*Expected product size is 260bp*

**Step 4: Illumina Sequencing Library Cleanup**

*(Adapted from AMpure XP manufacturer’s protocol)*

1. Add 1.8X volume of AMpureXP beads to each reaction (i.e. 90µl beads for a 50µl reaction) and incubate for 5 minutes at room temperature
2. Place tubes in the magnetic separation rack for at least 1 minute.
3. Without disturbing the beads on the side of the tube, pipette out the liquid leaving up to 20µl behind.
4. While still on the rack, add 200µl of 70% ethanol to each tube to wash the beads.
5. Incubate for 1 minute at room temperature.
6. Pipette out all the liquid from each tube.
7. Repeat Steps 4 to 6.
8. Air dry the beads for 2 minutes at room temperature
9. Remove tubes from rack and add 40µl of water/elution buffer to the beads, pipetting up and down to ensure all the beads are suspended.
10. Incubate at room temperature for a minimum of 5 minutes.
11. Place tubes in magnetic separation rack and let it sit for 5 minutes.
12. Without disturbing the beads on the side of the tube, pipette out the 40µl of liquid and put into a new tube.
13. Quantify the concentration of DNA using a spectrophotometer.

**Note: Different samples can be pooled to produce a pool with an equal amount of DNA per sample and run on one Illumina HiSeq/MiSeq sequencing lane if they are uniquely indexed.*
