# Supplementary material for: Multiplex shRNA Screening of Germ Cell Development by in Vivo Transfection of Mouse Testis
Source: G3 (Bethesda). 2016 Nov 15;7(1):247–55. doi: 10.1534/g3.116.036087 (PMC5217113; doi:10.1534/g3.116.036087)
Supplement: Supplementary file 8 [file 247FileS4.docx]

**SUPPLEMENTAL METHODS**

Here we discuss in additional detail some FAQ that we encountered during presentation of this work and the review of the manuscript

**WHY ARE SOME POSITIVE CONTROL CONSTRUCTS APPARENTLY ENRICHED IN THE PILOT SCREEN?**

In Figure 4A there are data for 83 clones targeting 17 unique genes known to play roles in spermatogenesis. To be clear we exclude the two motility genes from this list (SIRT1 and VDAC3). Only 14/83 (17%) of these clone show significant enrichment compared to the control clones. While we have clarified the number of clones with statistically significant enrichment, the reviewer may also wonder why any of the “positive controls” shows enrichment at all. The primary explanation is that this apparent “enrichment” is a necessary result of our normalization process. Due to variation in our already low transfection efficiency, we cannot simply count the abundance of each clone in testicular cells and compare the absolute counts to the input (if we did this, all of our clones would appear depleted all of the time). We normalize the counts to the total number of clones recovered. The following figure illustrates how, when we convert from absolute to relative abundance, even positive controls with deleterious effects can appear to be enriched post-testis:

Here, we have injected three clones at 50 copies each – 2 positive controls and one negative. They have a relative abundance of 30% in the input pool. The transfection efficiency is 20%, so 10 copies of each integrate. Positive control 1 has a 20% selective disadvantage, and thus 20% of cells with this construct die. Positive control 2 has an 80% selective disadvantage. Negative control has no selective effect. When we normalize the post-testis clones, Positive control 1 has actually increased in relative abundance, from 33% to 40%, despite being deleterious for germ cells.

**How did you settle on the N2a study design? Are your results biologically comparable to the testis screen?**

The doubling time of N2a cells has widely been reported to be approximately 24 hours (Klohn *et al.* 2003; Wicki-Stordeur *et al.* 2012). Furthermore, a previous study using shRNA transfection of N2a cells used 48 hours as the time-to-harvest, and found numerous and reproducible differences in cell physiology and mRNA splicing after 48 hours, as measured by RNAseq (Raj *et al.* 2011). We believe that 48 hours is sufficient to see selective effects (for instance, if an shRNA confers a 50% probability of cell death per cell cycle, one would only expect ~25% of the cell infected with that construct to remain after 48 hours).

Nonetheless, we agree with the reviewer that a longer N2a selection experiment would be a better comparison to the testis experiment, and thus we have performed extensive additional selection experiments for this revision. It has been estimated that there are approximately 10 cell divisions necessary to transform a murine SSC into a mature sperm. See for instance figure 5 from Drost and Lee (1995).

We say approximate because there are some inconsistencies in whether people consider the equational division after MII a “cell division” (it is not marked as one in this figure) as well as whether or not to count the SSC division producing A paired cells toward the total. At most there are 11 cell divisions based between A_s_ and sperm as indicated in the figure above.

Thus, we have cultured the N2a cells as before, this time, running the experiment for 12 days and 5 passages, sampling the cells at each passage. This should give us approximately 11 cell divisions during the course of the experiment. We performed this experiment in triplicate for each shRNA library, leading to the generation of 15 shRNA sequencing libraries, which we sequenced across two lanes of an Illumina MiSeq machine.

**References**

Boettcher, M., and Hoheisel, J.D. (2010). Pooled RNAi Screens - Technical and Biological Aspects. Curr. Genomics *11*, 162–167.

Echeverri, C.J., Beachy, P.A., Baum, B., Boutros, M., Buchholz, F., Chanda, S.K., Downward, J., Ellenberg, J., Fraser, A.G., Hacohen, N., et al. (2006). Minimizing the risk of reporting false positives in large-scale RNAi screens. Nat. Methods *3*, 777–779.

Galeev, R., Baudet, A., Kumar, P., Rundberg Nilsson, A., Nilsson, B., Soneji, S., Törngren, T., Borg, Å., Kvist, A., and Larsson, J. (2016). Genome-wide RNAi Screen Identifies Cohesin Genes as Modifiers of Renewal and Differentiation in Human HSCs. Cell Rep. *14*, 2988–3000.

Mohr, S.E., Smith, J.A., Shamu, C.E., Neumüller, R.A., and Perrimon, N. (2014). RNAi screening comes of age: improved techniques and complementary approaches. Nat. Rev. Mol. Cell Biol. *15*, 591–600.

Moindrot, B., Cerase, A., Coker, H., Masui, O., Grijzenhout, A., Pintacuda, G., Schermelleh, L., Nesterova, T.B., and Brockdorff, N. (2015). A Pooled shRNA Screen Identifies Rbm15, Spen, and Wtap as Factors Required for Xist RNA-Mediated Silencing. Cell Rep. *12*, 562–572.

Rudalska, R., Dauch, D., Longerich, T., McJunkin, K., Wuestefeld, T., Kang, T.-W., Hohmeyer, A., Pesic, M., Leibold, J., von Thun, A., et al. (2014). In vivo RNAi screening identifies a mechanism of sorafenib resistance in liver cancer. Nat. Med. *20*, 1138–1146.

Sims, D., Mendes-Pereira, A.M., Frankum, J., Burgess, D., Cerone, M.-A., Lombardelli, C., Mitsopoulos, C., Hakas, J., Murugaesu, N., Isacke, C.M., et al. (2011). High-throughput RNA interference screening using pooled shRNA libraries and next generation sequencing. Genome Biol. *12*, R104.

Zuber, J., Shi, J., Wang, E., Rappaport, A.R., Herrmann, H., Sison, E.A., Magoon, D., Qi, J., Blatt, K., Wunderlich, M., et al. (2011). RNAi screen identifies Brd4 as a therapeutic target in acute myeloid leukaemia. Nature *478*, 524–528.

Klohn, P. C., L. Stoltze, E. Flechsig, M. Enari and C. Weissmann, 2003 A quantitative, highly sensitive cell-based infectivity assay for mouse scrapie prions. Proc Natl Acad Sci U S A 100**:** 11666-11671.

Raj, B., D. O'Hanlon, J. P. Vessey, Q. Pan, D. Ray *et al.*, 2011 Cross-regulation between an alternative splicing activator and a transcription repressor controls neurogenesis. Mol Cell 43**:** 843-850.

Wicki-Stordeur, L. E., A. D. Dzugalo, R. M. Swansburg, J. M. Suits and L. A. Swayne, 2012 Pannexin 1 regulates postnatal neural stem and progenitor cell proliferation. Neural Dev 7**:** 11.
