# Supplementary material for: Multiplex shRNA Screening of Germ Cell Development by in Vivo Transfection of Mouse Testis
Source: G3 (Bethesda). 2016 Nov 15;7(1):247–55. doi: 10.1534/g3.116.036087 (PMC5217113; doi:10.1534/g3.116.036087)
Supplement: Supplementary file 11 [file 247TableS2.docx]

| **Sample** | **Mean**  **shRNA qPCR cycle** | **Standard Deviation**  **shRNA qPCR cycle** | **Mean**  **Actin qPCR cycle** | **Standard Deviation**  **Actin qPCR cycle** | **Transfection**  **Rate** |
| --- | --- | --- | --- | --- | --- |
| lentivirus 1 shot rep1 | 25.48693275 | 0.980911136 | 15.87373199 | 0.650952438 | 0.001229082 |
| lentivirus 1 shot rep2 | 30.67140961 | 0.239385545 | 20.69246101 | 0.140888497 | 0.000953849 |
|  |  |  |  |  |  |
| DNA 1 shot rep1 | 29.62903252 | 0.755530495 | 16.19665559 | 0.961291686 | 0.0000870752 |
| DNA 1 shot rep2 | 34.09828949 | 0.743369937 | 22.191576 | 0.202929765 | 0.000250706 |
|  |  |  |  |  |  |
| DNA 5 shots rep1 | 26.41898537 | 0.645285487 | 20.50484753 | 0.522136739 | 0.015962821 |
| DNA 5 shots rep2 | 25.82338905 | 0.411470205 | 21.09821939 | 0.416632252 | 0.036393577 |
| DNA 5 shots rep3 | 26.44046402 | 0.415599784 | 19.91761208 | 0.464417369 | 0.010468114 |
| DNA 5 shots rep4 | 25.54398956 | 0.581989333 | 19.63961601 | 0.557033598 | 0.016071226 |

**Table S2: Infection Rates for various injection conditions**

Each sample was prepared using at least 3 qPCR replicates for each target (Actin, shRNA). The formula to calculate the transfection rate was

$$\frac{1}{2^{shRNA cycle count- Actin cycle count}} \times\frac{Actin Primer efficiency}{shRNA Primer efficiency}$$

Assuming each cell has 1 copy of actin (haploid germ cell), this should provide the transfection rate of the shRNA in the test.
