# Supplementary material for: Multiplex shRNA Screening of Germ Cell Development by in Vivo Transfection of Mouse Testis
Source: G3 (Bethesda). 2016 Nov 15;7(1):247–55. doi: 10.1534/g3.116.036087 (PMC5217113; doi:10.1534/g3.116.036087)
Supplement: Supplementary file 14 [file 247TableS4.docx]

Table S4. Summary results of the predicted genes screen. (.txt, 5 KB)

<http://www.g3journal.org/lookup/suppl/doi:10.1534/g3.116.036087/-/DC1/TableS4.txt>
